# Role of Pellino-1 in Inflammation and Cardioprotection following Severe Sepsis: A Novel Mechanism in a Murine Severe Sepsis Model †

**DOI:** 10.3390/cells12111527

**Published:** 2023-06-01

**Authors:** Mahesh Thirunavukkarasu, Santosh Swaminathan, Andrew Kemerley, Seetur R. Pradeep, Sue Ting Lim, Diego Accorsi, Rickesha Wilson, Jacob Campbell, Ibnalwalid Saad, Siu-Pok Yee, J. Alexander Palesty, David W. McFadden, Nilanjana Maulik

**Affiliations:** 1 Department of Surgery, University of Connecticut School of Medicine, Farmington, CT 06032, USA; 2 Molecular Cardiology and Angiogenesis Laboratory, University of Connecticut School of Medicine, Farmington, CT 06032, USA; 3 Stanley J. Dudrick, Department of Surgery, Saint Mary’s Hospital, Waterbury, CT 06706, USA; 4 Center for Mouse Genome Modification, University of Connecticut Health School of Medicine, Farmington, CT 06032, USA

**Keywords:** septic shock, inflammation, cardiac function, infection, Peli1

## Abstract

Objectives: Intra-abdominal sepsis is commonly diagnosed in the surgical population and remains the second most common cause of sepsis overall. Sepsis-related mortality remains a significant burden in the intensive care unit despite advances in critical care. Nearly a quarter of the deaths in people with heart failure are caused by sepsis. We have observed that overexpression of mammalian Pellino-1 (Peli1), an E3 ubiquitin ligase, causes inhibition of apoptosis, oxidative stress, and preservation of cardiac function in a myocardial infarction model. Given these manifold applications, we investigated the role of Peli1 in sepsis using transgenic and knockout mouse models specific to this protein. Therefore, we aimed to explore further the myocardial dysfunction seen in sepsis through its relation to the Peli 1 protein by using the loss of function and gain-of-function strategy. Methods: A series of genetic animals were created to understand the role of Peli1 in sepsis and the preservation of heart function. Wild-type, global Peli1 knock out (Peli1^−/−^), cardiomyocyte-specific Peli1 deletion (CP1KO), and cardiomyocyte-specific Peli1 overexpressing (alpha MHC (αMHC) Peli1; AMPEL1^Tg/+^) animals were divided into sham and cecal ligation and puncture (CLP) surgical procedure groups. Cardiac function was determined by two-dimensional echocardiography pre-surgery and at 6- and 24-h post-surgery. Serum IL-6 and TNF-alpha levels (ELISA) (6 h), cardiac apoptosis (TUNEL assay), and Bax expression (24 h) post-surgery were measured. Results are expressed as mean ± S.E.M. Results: AMPEL1^Tg/+^ prevents sepsis-induced cardiac dysfunction assessed by echocardiographic analysis, whereas global and cardiomyocyte-specific deletion of Peli1 shows significant deterioration of cardiac functions. Cardiac function was similar across the sham groups in all three genetically modified mice. ELISA assay displayed how Peli 1 overexpression decreased cardo-suppressive circulating inflammatory cytokines (TNF-alpha, IL-6) compared to both the knockout groups. The proportion of TUNEL-positive cells varied according to Peli1 expression, with overexpression (AMPEL1^Tg/+^) leading to a significant reduction and Peli1 gene knockout (Peli1^−/−^ and CP1KO) leading to a significant increase in their presence. A similar trend was also observed with Bax protein expression. The improved cellular survival associated with Peli1 overexpression was again shown with the reduction of oxidative stress marker 4-Hydroxy-2-Nonenal (4-HNE). Conclusion: Our results indicate that overexpression of Peli1 is a novel approach that not only preserved cardiac function but reduced inflammatory markers and apoptosis following severe sepsis in a murine genetic model.

## 1. Introduction

Sepsis is defined as a systemic deleterious host response to infection, which can lead to severe sepsis (acute organ dysfunction) and septic shock (severe sepsis with refractory hypotension) [1]. The morbidity and mortality from sepsis and its sequelae remain a significant burden to the American healthcare system despite ongoing advances in critical care. These advances include the Surviving Sepsis campaign, which helped to protocolize the treatment of this severe disease process and reduce overall hospital mortality from 37 to 30.8% over two years [2]. Although the effects of sepsis are widespread, the cardiovascular system is of particular importance, given the resultant increase in mortality when it is affected [3]. Sepsis-induced myocardial dysfunction (SIMD) has significant variations in its reported prevalence, a finding that may result from the lack of a universal definition of SIMD. The typical clinical manifestations include impaired systolic contractility and diastolic function [4]. Factors involved in the pathogenesis of SIMD potentially include activation of pathogen-associated molecular patterns (PAMPs) and Toll-like receptors (TLRs), tissue injury from danger-associated molecular patterns (DAMPs), elevated levels of cytokines such as tumor necrosis factor-alpha (TNF-α), presence of reactive oxygen species, mitochondrial dysfunction, and myocardial calcium dysregulation.

This study describes the role of Pellino-1 (Peli1) in sepsis and functions to determine its potential benefit as a target for ameliorating SIMD in severe sepsis. Pellino is a conserved scaffold protein, first identified in *Drosophila melanogaster* as an interaction partner of the Drosophila Pelle kinase, an ortholog of the Interleukin-1 receptor-associated kinase. Four unique forms (Pellino-1, Pellino-2, Pellino-3A, and Pellino-3B) have been isolated thus far [5,6,7,8,9,10]. Our laboratory has thoroughly studied the role of the Peli1 protein as a mediator of angiogenesis and ischemic remodeling following myocardial infarction and hind limb ischemia [11,12]. We have demonstrated in a prior study that Peli1 gene therapy has benefits after reconstructive surgery in decreasing necrosis of ischemic skin flaps [13]. In addition, we have extensively studied its impact on post-ischemic recovery in myocardial infarction and hind limb ischemia models and its role in modulating oxidative stress and anti-apoptotic properties [11]. Pellino-1 overexpression has been shown to counteract oxidative stress from peroxide-induced damage to cells and triggers cIAP2, an essential anti-apoptotic protein that inhibits caspases 3, 7, and 9. Significantly uncontrolled inflammatory immune response triggers differential protein expression in various signaling pathway components that involve the cardiovascular, neurological, and endocrine systems, energy metabolism, and so on, which leads to triggering death signal (cell death), mitochondrial dysfunction, ischemia/hypoxia, and ultimately multi-organ dysfunction (MOD) [14]. 

In this study, severe intra-abdominal sepsis was induced in all the animals via the well-established and reliable model of cecal ligation and puncture (CLP) in order to evaluate the anti-apoptotic, anti-inflammatory role of Peli1, along with cardiac function using three different genetically modified mouse colonies related to Peli1.

## 2. Materials and Methods

### 2.1. Animal Studies

All experiments involving animals, including the surgeries performed, have been approved by the Institutional Animal Care and Use Committee (IACUC) of the University of Connecticut Health (Farmington, CT, USA). (IACUC protocol number: AP-200171-1023).

Animals: In the present study, three types of genetically modified mice were used: (1) cardiomyocyte-specific alpha-MHC Peli1 transgenic (AMPEL1^Tg/+^), (2) Peli1 global knockout mice (Peli1^−/−^ or Peli1KO), and (3) cardiomyocyte-specific Peli1 knockout (CP1KO); the controls for each type were C57BL/6J (WT) mice for AMPEL1^Tg/+^ and Peli1^fl/fl^ (WT) for Peli1^−/−^ and CP1KO, respectively.

#### 2.1.1. Generation of Peli1^fl/fl^ and Peli1KO mice 

The details of Peli1^fl/fl^ and Peli1 global knockout mice are described in our earlier publication [11]. 

#### 2.1.2. Generation of Cardiomyocyte-Specific Peli1 (CP1KO) Knockout Mice

Peli1 targeting vector design and generation of chimeric mice were performed according to Liu et al. [15]. Chimeric mice with LoxP sites flanking exons 3 and 4 of Peli1 and Frt-flanked PGKneo drug-selectable markers were bred with Rosa26-flpe (Jax 003946) to remove the PGKneo cassette to generate Peli1^fl/+^ mice. These floxed mice were subsequently bred with α-MHC Cre (Jax 011038) to generate the final cardiomyocyte-specific Peli1 knockout mice. The primer pair, LoxgtF (5′-ACT CCA TTT CCC TCA AAT GC) and LoxgtR (3′-ACA CCC AGG AAG CAC TGA AC), amplified fragments of 252bp and 307bp specific to the wild-type and 5’LoxP site, respectively, and the primer pair, LoxgtF and FrtgtR (5′-GGG AGG AAA GGG TTT ATT CG) were responsible for the amplification of the 365bp fragment specific to the Peli1 knockout allele.

#### 2.1.3. Generation of AMPEL1^Tg/+^ (Cardiomyocytes Overexpressing Peli1)

A 5.4 kb quantity of α-MHC promoter fragment was isolated from Alpha-MyHC clone 26 plasmid (a generous gift from Jeffrey Robbins). This promoter fragment was placed 5′ of Peli1 coding sequence, followed by the SV40 polyadenylation signal sequence. The transgene was released from the plasmid, purified, and microinjected into the pronucleus of one-cell C57BL6 embryos to generate the cardiomyocyte-overexpressing Peli1 (AMPELl^Tg/+^) mice. The primer pair, MHC 5F (5′-CAC TGT GGT GCC TCG TTC CAG C) and Peli1 5R (5′-GGC AGC CTG AGG AGT ACA TGC), which will amplify a fragment of 587 bp specific to the transgene, were used to genotype AMPEL1^Tg/+^ mice.

### 2.2. Study Design

**Figure 1:**
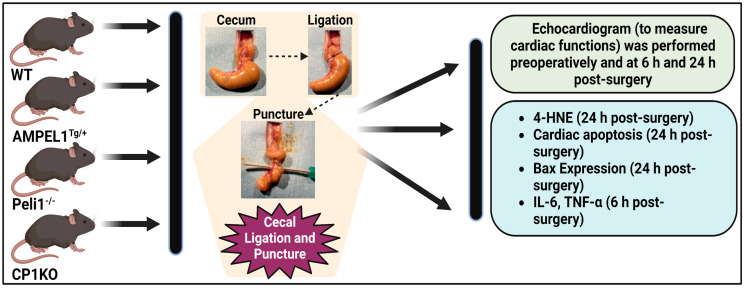
Schematic view of the experimental design. WT, AMPEL1^Tg/+^, Peli1^−/−^, and CP1KO mice aged 8–12 weeks were subjected to cecal ligation and puncture to create a sepsis model. In the sham groups, all animals underwent surgery without cecal ligation and puncture. The experiment was divided into pre- and post-CLP (6 h and 24 h). Echocardiographic analysis was performed preoperatively and post-surgery at 6 h and 24 h. Blood was collected 6 h post-surgery for the analysis of IL–6 and TNF–α by ELISA. All animals were sacrificed (24 h), and heart tissues were collected for immunohistochemical analysis.

Briefly, male and female mice 8–12 weeks old were used throughout the study. In order to understand the role of specific Peli1 gene deletion or addition within cardiomyocytes or all somatic cells during severe sepsis, the animals in each group were randomly categorized into sham and CLP groups in each genetically modified animal (AMPEL1^Tg/+^, Peli1^−/−^ and CP1KO animals) and subdivided into Experiments 1, 2, and 3, which are described below (Figure 1).

#### 2.2.1. Experiment 1: Effect of Cardiomyocyte Overexpression of Peli1 (AMPELl^Tg/+^) in Mice Subjected to CLP

The first part of our experiment aimed to assess the effects of cardiomyocyte overexpression of Peli1 in mice undergoing CLP. AMPELl^Tg/+^ mice and their respective wild-type (WT) controls were subjected to CLP in the following groups: WT sham (WTS), AMPELl^Tg/+^ sham (AMPEL1^Tg/+^S), WTCLP, and AMPELl^Tg/+^CLP.

#### 2.2.2. Experiment 2: Effect of Peli1 Gene Knockout in Mice Subjected to CLP

The second part of our experiment assessed the effects of global Peli1 gene knockout in mice undergoing CLP. Peli1 KO and their respective WT controls were subjected to CLP in the following groups: WT sham (WTS), Peli1^−/−^ sham (Peli1^−/−^S), WTCLP, and Peli1^−/−^CLP. 

#### 2.2.3. Experiment 3: Effect of Cardiomyocyte-Specific Peli1 (CP1KO) Knockout in Mice Subjected to CLP

The final part of our experiment focused on assessing the effects of cardiomyocyte-specific Peli1 knockout in mice undergoing CLP. CP1KO and their respective WT controls were subjected to CLP in the following groups: WT sham (WTS), CP1KO sham (CP1KOS), WTCLP, and CP1KOCLP.

For experiments 1, 2, and 3, functional parameters were obtained at predetermined time points using an echocardiogram. Tissues obtained from experiments 1, 2, and 3 were examined for the extent of myocardial apoptosis and 4-hydroxynonenal (4-HNE) and Bax expression. Blood samples collected were examined for inflammatory cytokine levels.

#### 2.2.4. Murine Cecal Ligation and Puncture Model (CLP Model)

Animals were first anesthetized by intraperitoneal injections of ketamine (100 mg/kg body weight) and xylazine (10 mg/kg of body weight). Following confirmation of adequate anesthesia by the absence of a toe pinch reflex, ventral abdominal hair was removed, and the underlying skin was prepped with ethanol and betadine preparations. A midline vertical skin incision of about 2 to 3 cm was then made, the underlying fascia was opened, and the peritoneal cavity entered. The cecum was successfully identified, externalized, and ligated distal to the ileocecal valve using a free silk tie, leaving about 75% distal to the point of ligation. The cecal contents were milked towards the blind end prior to the ligation. Following this, an 18-gauge needle was then used to create two through-and-through punctures, which we have earlier demonstrated to yield results in severe sepsis [8]. A small amount of fecal content was expressed from these perforations, confirming the presence of the two through-and-through punctures, and the cecum was returned to its intraperitoneal position. The fascia was closed in a running fashion using 5-0 Vicryl sutures with care to avoid including the bowel in the closure, and the skin closed in a simple interrupted fashion with 5-0 Vicryl. Fluid resuscitation in the form of pre-warmed 0.9% normal saline was injected intraperitoneally post-closure along with analgesia in the form of subcutaneous buprenorphine (0.05–0.1 mg/kg). The animals were monitored in a recovery bin with adequate heating and light. After recovery from anesthesia, they were returned to their cages. Subsequently, analgesia in the form of subcutaneous buprenorphine (0.05–0.1 mg/kg) was administered at serial intervals of 12 h. The sham procedure involved the described steps, including skin incision, exploration of the abdominal cavity with externalization of the cecum, and subsequent return to the abdomen without ligation or puncture. Steps including fluid administration and analgesia were performed as a part of the sham procedure [16].

### 2.3. Echocardiography 

Echocardiography during the study was performed preoperatively at 6- and 24-h time points using the Vevo 770, 2100, and the 3100 micro-imaging system (FUJIFILM VisualSonics, Inc., Toronto, ON, Canada). Following inhalational anesthesia (induction and maintenance) using 2% isoflurane and an oxygen flow rate of 1.5 L/min, images were obtained from the parasternal long axis and short axis views, and subsequent measurements of various echocardiographic variables were performed using the VevoLAB software 3.2.5 [11,12,16]. 

#### 2.3.1. Immunohistochemical Analysis of 4-Hydroxynonenal (4-HNE) Expression

Immunohistochemical analysis of 4-HNE was performed in cardiac sections collected at the 24-h time point. Five-μm-thick sections of cardiac tissue were incubated with primary antibody anti-4 hydroxynonenal (HNE) anti-serum (Cat# HNE11-S; Alpha Diagnostic International Inc., San Antonis, TX, USA), followed by the ImmPRESS Horse Anti-Rabbit IgG Polymer kit, Peroxidase (Cat# MP-7401, Vector Laboratories, Inc., Newark, CA, USA). Avidin-biotin peroxidase (ABC) complex (Vectastain Elite ABC Kit; Vector Laboratories) was used with HRP-DAB to visualize antigen–antibody immunoreaction. Sections were counterstained with Mayer’s hematoxylin to stain nuclei, and the images were captured at 40× magnification using the Olympus BH2 microscope [11].

#### 2.3.2. Immunofluorescence Analysis of Bax Expression

In this study, five-μm cardiac sections collected at the 24-h time point were incubated with primary rabbit antibody (Cat# Sc-493; Santa Cruz Biotechnology Inc., Dallas, TX, USA), followed by Alexa Fluor 555 secondary antibody (Cat # A21428; ThermoFisher scientific, Waltham, MA, USA). Sections were mounted with UltraCruz Aqueous Mounting Medium with DAPI (Sc-24941; Santa Cruz Biotechnology, Inc., Dallas, TX, USA). Images were taken at 20× magnification using an IRIS digital imaging system (Logos Biosystems, Annandale, VA, USA) [12,17,18]. 

#### 2.3.3. TUNEL Apoptosis Assay

In brief, the terminal deoxynucleotidyl transferase dUTP nick-end labeling (TUNEL) method was employed using the commercially available in situ Cell Death Detection Kit, Fluorescein (11684795910, Roche, Mannheim, Germany) to quantify the apoptosis in cardiac tissue at the 24-h time point. Sections were mounted with UltraCruz Aqueous Mounting Medium with DAPI (Sc-24941; Santa Cruz Biotechnology Inc., Dallas, TX, USA), and images were taken at 20× using IRIS digital imaging system (Logos Biosystems, Annandale, VA, USA). For the quantification of TUNEL-positive cells, approximately 8 fields per section were examined and expressed in the percentage of TUNEL-positive cells relative to the total cell number (nuclei) [16,19].

#### 2.3.4. ELISA to Measure Serum Cytokines 

Blood was collected post-procedure at the 6-h time point and centrifuged at 2500 rpm for 15 min at 4 °C to collect the serum, and the level of circulating cytokines such as Tumor Necrosis Factor-alpha (TNF-α; Cat# 430907) and Interleukin-6 (IL-6; Cat# 431307, BioLegend, San Diego, CA, USA) were measured using commercially available kits per the manufacturer’s instructions.

**Figure 2:**
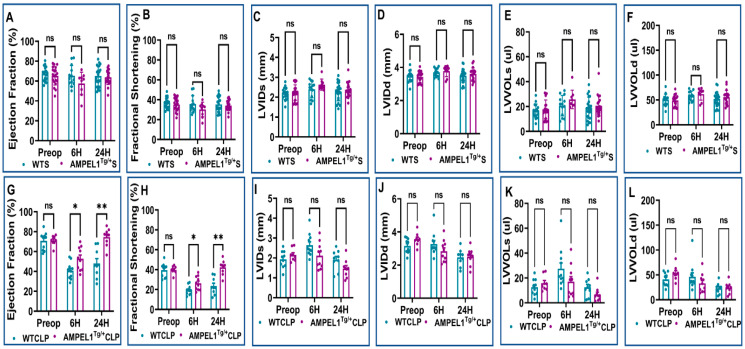
Graphical representation of preoperative, 6 h, and 24 h echocardiographic analysis comparing wild-type sham (WTS) to AMPEL1^Tg/+^ S: (**A**) ejection fraction (EF), (**B**) fractional shortening (FS), (**C**) left ventricular systolic internal diameter (LVIDs), (**D**) left ventricular diastolic internal diameter (LVIDd), (**E**) left ventricular volume at systole (LVVOLs), (**F**) left ventricular volume at diastole (LVVOLd); comparing wild-type CLP and AMPEL1^Tg/^ CLP: (**G**) ejection fraction (EF), (**H**) fractional shortening (FS), (**I**) left ventricular systolic internal diameter (LVIDs), (**J**) left ventricular diastolic internal diameter (LVIDd), (**K**) left ventricular volume at systole (LVVOLs), (**L**) left ventricular volume at diastole (LVVOLd). Values are mean ± S.E.M.: (WT sham: *n* = 13–22); (WT CLP: *n* = 9–11); (AMPEL1^Tg/+^ sham: *n* = 9–20); (AMPEL1^Tg/+^ CLP: *n* = 8–10). Where ns-not significant; * *p* < 0.05, ** *p* < 0.01.

**Figure 3:**
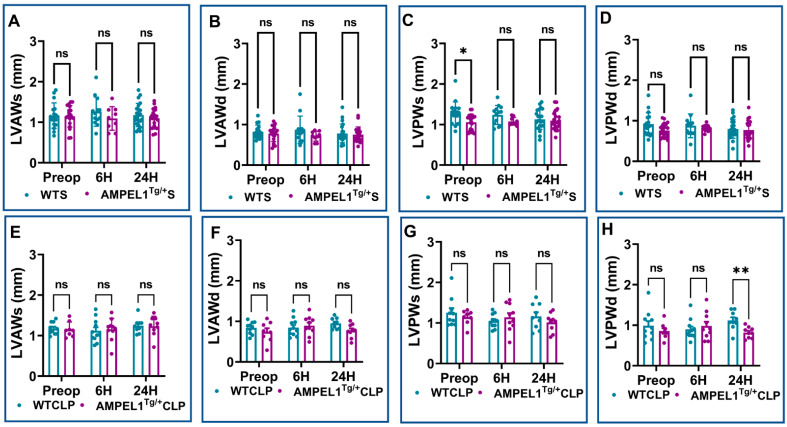
Graphical representation of preoperative, 6 h, and 24 h echocardiographic analysis comparing wild-type sham (WTS) to AMPEL1^Tg/+^ S: (**A**) left ventricular anterior wall thickness at systole (LVAWs), (**B**) left ventricular anterior wall thickness at diastole (LVAWd), (**C**) left ventricular posterior wall thickness at systole (LVPWs), (**D**) left ventricular posterior wall thickness at diastole (LVPWd); comparing wild-type CLP and AMPEL1^Tg/+^CLP: (**E**) left ventricular anterior wall thickness at systole (LVAWs), (**F**) left ventricular anterior wall thickness at diastole (LVAWd), (**G**) left ventricular posterior wall thickness at systole (LVPWs), (**H**) left ventricular posterior wall thickness at diastole (LVPWd). Values are mean ± S.E.M.: (WTS: *n* = 13–22); (WT CLP: *n* = 9–11); (AMPEL1^Tg/+^ S: *n* = 9–20); (AMPEL1^Tg/+^ CLP: *n* = 8–10). Where ns-not significant; * *p* < 0.05, ** *p* < 0.01.

**Figure 4:**
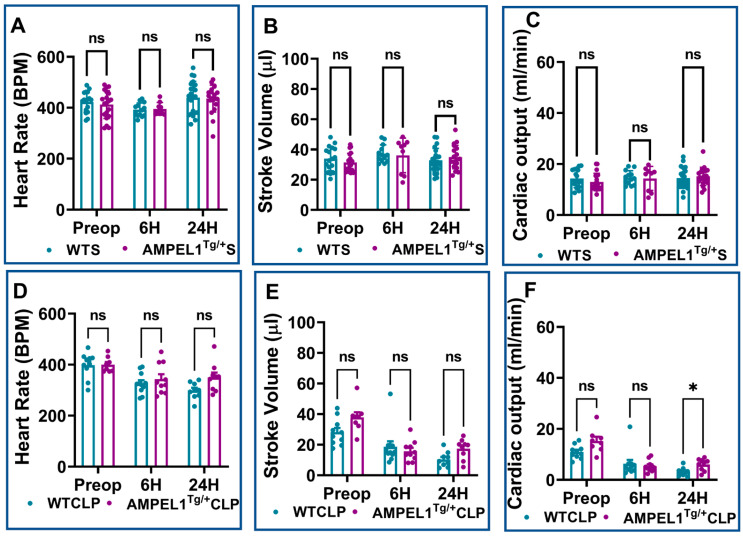
Graphical representation of preoperative, 6 h, and 24 h echocardiographic analysis comparing wild-type sham (WTS) to AMPEL1^Tg/+^ S: (**A**) heart rate (HR), (**B**) stroke volume (SV), (**C**) cardiac output (CO); comparing wild-type CLP and AMPEL1^Tg/+^CLP: (**D**) heart rate (HR), (**E**) stroke volume (SV), (**F**) cardiac output (CO). Values are mean ± S.E.M.: (WTS: *n* = 13–22); (WT CLP: *n* = 9–11); (AMPEL1^Tg/+^ S: *n* = 9–20); (AMPEL1^Tg/+^ CLP: *n* = 8–10). Where ns-not significant; * *p* < 0.05.

**Figure 5:**
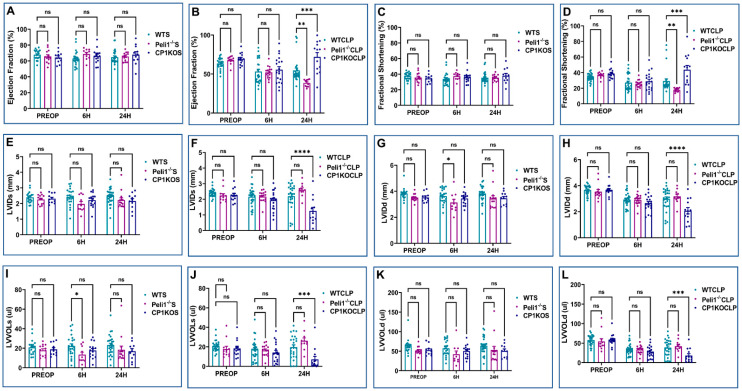
Graphical representation of preoperative, 6 h, and 24 h echocardiographic analysis comparing wild-type sham, wild-type CLP, Peli1^−/−^ sham, Peli1^−/−^ CLP, CP1KO sham, and CP1KO CLP: (**A**,**B**) ejection fraction (EF) of wild-type sham (WTS) and CLP, (**C**,**D**) fractional shortening (FS) of WT sham (WTS) and CLP, (**E**,**F**) left ventricular systolic internal diameter (LVIDs) of sham (S) and CLP, (**G**,**H**) left ventricular diastolic internal diameter (LVIDd) of sham (S) and CLP, (**I**,**J**) left ventricular volume at systole (LVVOLs) of sham (S) and CLP, (**K**,**L**) left ventricular volume at diastole (LVVOLd) of sham and CLP. Values are mean ± S.E.M.: (WTS: *n* = 19–28); (WT CLP: *n* = 21–27); (Peli1^−/−^S: *n* = 10–14); (Peli1^−/−^ CLP: *n* = 11–16); (CP1KOS: *n* = 11–15); (CP1KO CLP: *n* = 13–19). Where ns-not significant; * *p* < 0.05, ** *p* < 0.01, *** *p* < 0.001, **** *p* < 0.0001.

**Figure 6:**
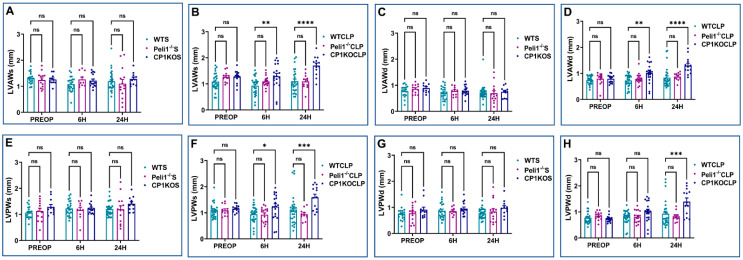
Graphical representation of preoperative, 6 h, and 24 h echocardiographic analysis comparing wild-type sham (WTS), WTCLP, Peli1^−/−^S, Peli1^−/−^ CLP, CP1KOS, and CP1KO CLP: (**A**,**B**) left ventricular anterior wall thickness at systole (LVAWs) of sham (S) and CLP, (**C**,**D**) left ventricular anterior wall thickness at diastole (LVAWd) of sham (S) and CLP, (**E**,**F**) left ventricular posterior wall thickness at systole (LVPWs) of sham and CLP, (**G**,**H**) left ventricular posterior wall thickness at diastole (LVPWd) of sham and CLP. Values are mean ± S.E.M.: (WTS: *n* = 19–28); (WT CLP: *n* = 21–27); (Peli1^−/−^ S: *n* = 10–14); (Peli1^−/−^ CLP: *n* = 11–16); (CP1KOS: *n* = 11–15); (CP1KO CLP: *n* = 13–19). Where ns-not significant; * *p* < 0.05, ** *p* < 0.01, *** *p* < 0.001, **** *p* < 0.0001.

**Figure 7:**
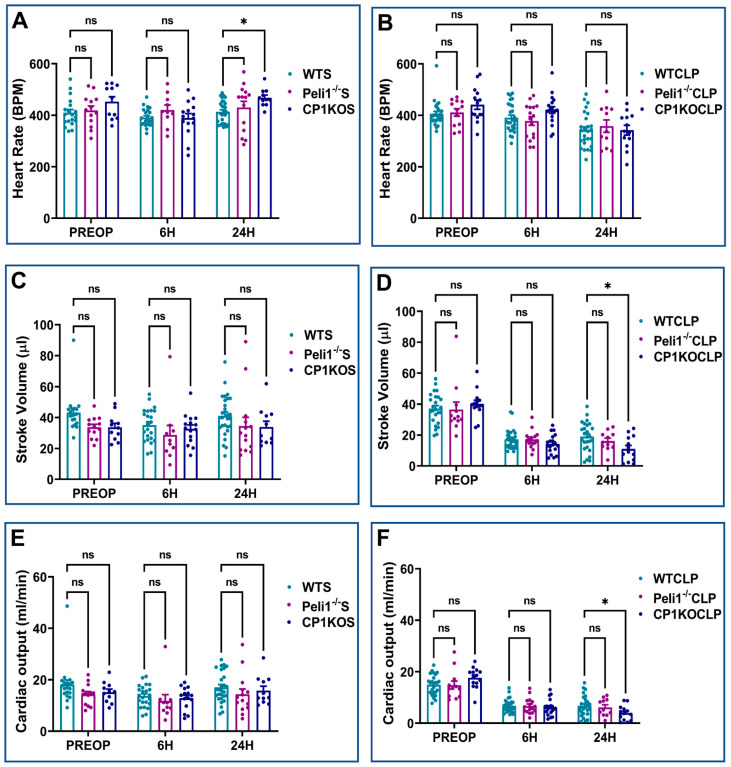
Graphical representation of preoperative, 6 h, and 24 h echocardiographic analysis comparing wild-type sham, wild-type CLP (WTCLP), Peli1^−/−^ S, Peli1^−/−^ CLP, CP1KOS, and CP1KO CLP: (**A**,**B**) heart rate (HR) of sham (S) and CLP, (**C**,**D**) stroke volume (SV) of sham (S) and CLP, (**E**,**F**) cardiac output (CO) of sham and CLP. Values are mean ± S.E.M., (WT sham: *n* = 19–28); (WT CLP: *n* = 21–27); (Peli1^−/−^ sham: *n* = 10–14); (Peli1^−/−^ CLP: *n* = 11–16); (CP1KO sham: *n* = 11–15); (CP1KO CLP: *n* = 13–19). Where ns-not significant; * *p* < 0.05.

Statistical analysis: GraphPad Prism software version 9 for Windows (GraphPad, San Diego, CA, USA) was used for statistical analysis. Values are expressed as mean ± standard error of the mean (S.E.M.). A multiple Mann–Whitney test was used to compare the differences between the two groups. For Figure 2, Figure 3 and Figure 4, multiple Mann–Whitney test were used and corrected for multiple comparisons using the Holm–Sidak method to assess significant differences between the groups. For Figure 5, Figure 6 and Figure 7, two-way ANOVA followed by Dunnett’s multiple comparisons test to assess significant differences between the groups. For Figure 9D, an unpaired *t*-test was used to compare the differences between the two groups. For Figure 10 way ANOVA followed by Tukey’s multiple comparisons test was used. A ‘P’ value less than 0.05 was considered statistically significant. 

## 3. Results 

### 3.1. Overexpression of Cardiomyocyte-Specific Pellino-1 (AMPEL1^Tg/+^) Allows Preservation of Ejection Fraction and Other Echocardiographic Parameters following Severe Sepsis

Figure 2, Figure 3 and Figure 4 demonstrate the various echocardiographic parameters following sham and CLP surgery in WT and AMPEL1^Tg/+^ mice at the various study time points (preoperative and 6 h and 24 h after surgery). Preoperative echocardiographic analysis showed no statistical difference between WTS and AMPEL1^Tg/+^S mice in the following measured parameters: EF, FS, LVIDs, LVIDd, LVVOLs, LVVOLd, LVAWs, LVAWd, LVPWd, HR, SV, and CO, respectively, except LVPWs (*n* = 17–20). Also, there is minimal to no significant difference between the two groups at 6 h and 24 h post-sham surgery. Graphical representations of these different parameters are shown in Figure 2A–F, Figure 3A–D and Figure 4A–C.

Similarly, Figure 2G–L, Figure 3E–H and Figure 4D–F represent various echocardiographic parameters measured 6 h and 24 h post-CLP surgery. WTCLP mice were found to have a statistically significant decrease in EF at 6 h (40.68 ± 2.46 vs. 70.67 ± 2.85; %; *n* = 10–11) and 24 h (48.04 ± 4.85 vs. 70.67 ± 2.85; %; n = 9–10) when compared to preoperative EF. This was also observed in EF in the AMPEL1^Tg/+^CLP mice at 6 h (52.74 ± 3.71 vs. 71.04 ± 1.81; %; *n* = 8–10) and 24 h (74.59 ± 2.96 vs. 71.04 ± 1.81; %; *n* = 8–9) post-CLP, when compared to preoperative levels. To summarize, mice in the AMPEL1^Tg/+^CLP group demonstrated a higher EF at both the 6 h (52.74 ± 3.71 vs. 40.68 ± 2.46; %; *n* = 10–11; *p* = 0.02) and 24 h (74.59 ± 2.96 vs. 48.04 ± 4.85; %; *n* = 9; *p* = 0.0008) time points when compared to WTCLP at the same respective time points (Figure 2G).

This trend continued to remain significant between the WTCLP [preoperative: (39.42 ± 2.23 vs. 39.67 ± 1.40; %; *n* = 8–10; *p* = 0.90); 6 h: (19.33 ± 1.37 vs. 26.30 ± 2.24; %; *n* = 10–11; *p* = 0.02), 24 h (23.42 ± 2.83 vs. 42.25 ± 2.39; %; *n* = 9; *p* = 0.0005)] and AMPEL1^Tg/+^CLP groups for the other echocardiographic parameter, i.e., fractional shortening (FS) (Figure 2H). We also observed a significant decrease in the AMPEL1^Tg/+^CLP group’s LVPWd (0.82 ± 0.05 vs. 1.12 ± 0.08; mm; *n* = 9; *p* = 0.003) and an increase in their CO (6.03 ± 0.79 vs. 3.15 ± 0.56; ml/min; *n* = 9; *p* = 0.01) at 24 h post-CLP compared to the WTCLP group. Other measured echocardiographic parameters such as LVIDs, LVIDd, LVVOLs, LVVOLd, LVAWs, LVAWd, LVPWs, HR, and SV showed no statistical difference when compared between WTCLP and AMPEL1^Tg/+^CLP groups (Figure 2I–L, Figure 3E–H and Figure 4D,E).

### 3.2. Effect of Pellino-1 Global Knockout (Peli1^−/−^) and Cardiomyocyte-Specific Pellino-1 Knockout (CP1KO) Mice Subjected to Severe Sepsis on Cardiac Functions

Echocardiographic analysis was performed on WT, Peli1^−/−^, and CP1KO mice before and after sham and CLP surgery. There was no significant difference between WT and Peli1^−/−^ mice preoperatively in the echo parameters measured (Figure 5, Figure 6 and Figure 7). Similarly, the same results were seen when the heart functions of WT and CP1KO mice were measured with an echocardiogram before surgery (Figure 5, Figure 6 and Figure 7). Post-operative echocardiographic analyses were performed at 6 h and 24 h post-sham and -CLP surgery in both Peli1^−/−^ and CP1KO mice and compared to the corresponding WT groups at similar time points.

All echocardiographic parameters measured at 6 h and 24 h post-sham surgery showed no significant changes between WTS and Peli1^−/−^S, except LVIDd (3.63 ± 0.12 vs. 3.13 ± 0.25; mm; *n* = 10–23; *p* = 0.04) and LVVOLs (22.26 ± 2.26 vs. 13.40 ± 2.60; µL; *n* = 10–23; *p* = 0.03), which showed significant decreases in the Peli1^−/−^S group compared to the WTS group at 6 h post-sham surgery (Figure 5A,C,E,I and Figure 6A). There were also no significant changes observed between the WTS and CP1KOS groups preoperatively or at post-operative time points 6 h and 24 h after sham surgery, except heart rate, which showed an increase in the CP1KOS group (467.92 ± 10.32 vs. 413.11 ± 8.19; bpm; *n* = 11–28; *p* = 0.02) at 24 h post-surgery compared to the WTS group (Figure 7A).

Echocardiographic analysis post-CLP surgery showed reduced EF, FS, LVIDd, LVVOLs, LVVOLd, SV, and CO in both the WTCLP and Peli1^−/−^CLP groups compared to their respective preoperative data (Figure 5, Figure 6 and Figure 7). There was no significant difference between WTCLP and Peli1^−/−^CLP in the echocardiographic parameters measured 6 h post-surgery. However, we observed significantly reduced EF (38.48 ± 1.36 vs. 54.55 ± 3.11; %; *n* = 11–26; *p* = 0.0013) and FS (18.04 ± 0.77 vs. 29.17 ± 2.88; %; *n* = 11–26; *p* = 0.007) at 24 h post-surgery in the Peli1^−/−^CLP group compared to the WTCLP group (Figure 5B,D). All other parameters measured (LVIDs, LVIDd, LVAWs, LVPWs, LVAWd, LVPWd, LVVOLs, LVVOLd, HR, SV, and CO) showed no significant difference between the WTCLP and Peli1^−/−^CLP groups (Figure 5, Figure 6 and Figure 7).

Similarly, echocardiography was also performed on both the WT mice and the CP1KO mice before and after the CLP surgery. When compared to data from before surgery, the results showed that both EF and FS were lower in both the WTCLP and CP1KOCLP groups at 6 h post-surgery. However, at 24 h post-surgery, both EF (71.89 ± 5.61 vs. 54.55 ± 3.11; %; *n* = 13–26; *p* = 0.0002) and FS (43.74 ± 5.53 vs. 29.16 ± 2.88; %; *n* = 13–26; *p* = 0.0001) were significantly increased in the CP1KOCLP group compared to the WTCLP group (Figure 5B,D). Correlating to this result, we also found significant reduction of LVIDs (1.25 ± 0.23 vs. 2.20 ± 0.16; mm; *n* = 13–26; *p* < 0.0001) and LVIDd (2.07 ± 0.24 vs. 2.97 ± 0.17; mm; *n* = 13–26; *p* < 0.0001) in the CP1KOCLP group compared to the WTCLP group at 24 h post-surgery. (Figure 5F,H). Similar results were found with LVVOLs (19.75 ± 2.33 vs. 7.07 ± 3.10; µL; *n* = 13–26; *p* = 0.0002) and LVVOLd (38.70 ± 4.09 vs. 18.10 ± 4.77; µL; *n* = 13–26; *p* = 0.0006) between the WTCLP and CP1KOCLP groups at 24 h post-surgery (Figure 5J,L). No significant difference was found between these groups for LVIDs, LVIDd, LVVOLs, and LVVOLd at 6 h post-surgery.

Consistent with our previous observations, wall dimension echocardiographic parameters showed a significant increase in wall thickness between WTCLP and CP1KOCLP groups. At 6 h after surgery, we found a statistical difference between the CP1KOCLP group and WTCLP group in terms of wall thickness parameters LVAWs (1.29 ± 0.11 vs. 0.93 ± 0.07; mm; *n* = 19–27; *p* = 0.0015), LVAWd (1.01 ± 0.08 vs. 0.72 ± 0.05; mm; *n* = 19–27; *p* = 0.0012), and LVPWs (1.24 ± 0.11 vs. 0.94 ± 0.06; mm; *n* = 19–27; *p* = 0.02) except LVPWd (1.01 ± 0.09 vs. 0.82 ± 0.05; mm; *n* = 19–27; *p* = 0.09). Similar data were observed in the CP1KOCLP and WTCLP groups at 24 h post-surgery in terms of wall dimensions [LVAWs (1.70 ± 0.10 vs. 1.10 ± 0.09; mm; *n* = 13–26; *p* < 0.0001), LVAWd (1.29 ± 0.09 vs. 0.83 ± 0.08; mm; *n* = 13–26; *p* < 0.0001), LVPWs (1.59 ± 0.13 vs. 1.12 ± 0.12; mm; *n* = 13–26; *p* = 0.0009), and LVPWd (1.40 ± 0.14 vs. 0.93 ± 0.10; mm; *n* = 13–26; *p* = 0.0001)] (Figure 6). 

**Figure 8:**
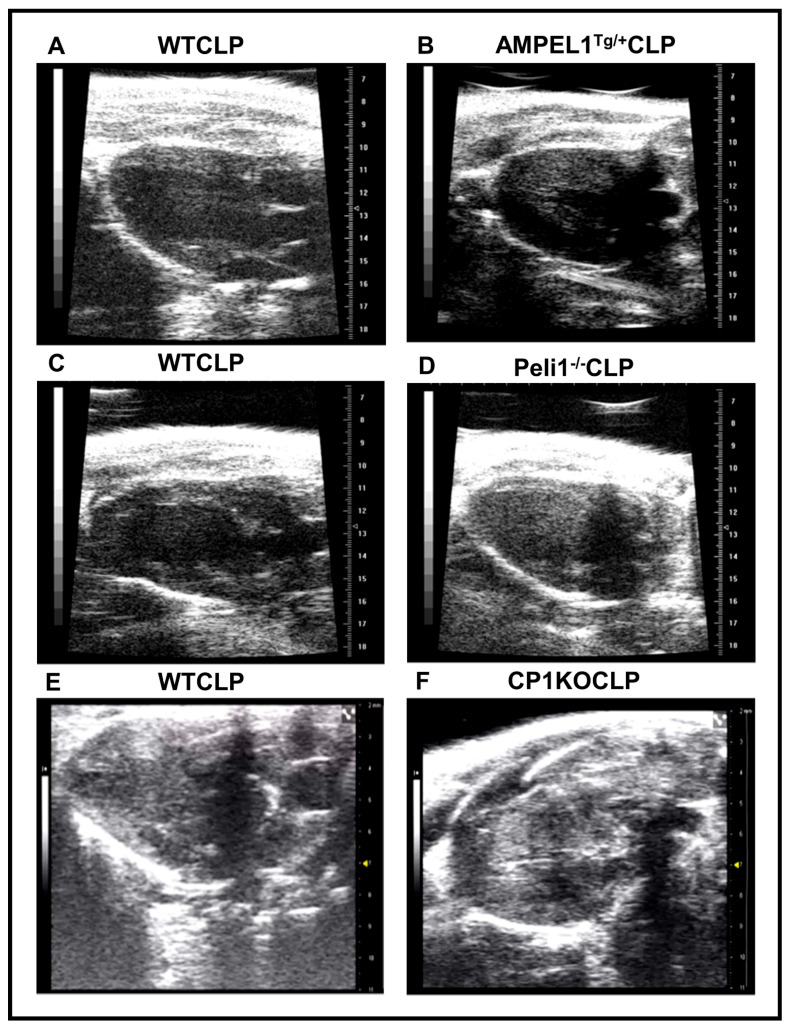
Representative echocardiographic images of long-axis view of WT, AMPEL1^Tg/+^, Peli1^−/−^ and CP1KO mice after 24 h post-CLP surgery. (**A**) Respective WTCLP; (**B**) AMPEL1^Tg/+^CLP; (**C**) Respective WTCLP; (**D**) Peli1^−/−^CLP; (**E**) Respective WTCLP; (**F**) CP1KOCLP images.

There was no significant difference in stroke volume and cardiac output at 6 h post-CLP. However, cardiac output (3.90 ± 0.80 vs. 6.72 ± 0.79; ml/min; *n* = 13–26; *p* = 0.05) and stroke volume (11.02 ± 2.24 vs. 18.95 ± 1.84; µL; *n* = 13–26; *p* = 0.02) was significantly reduced in the CP1KOCLP group compared to the WTCLP group at 24 h post-surgery (Figure 7. Representative B-mode images of parasternal long-axis views are provided in Figure 8.

### 3.3. Assessment of 4-HNE Expression following Sepsis 

**Figure 9:**
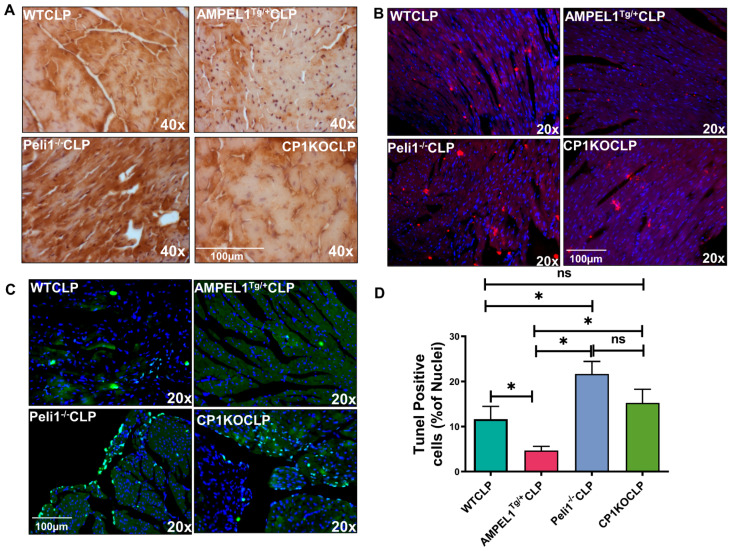
Immunohistochemical analysis of hearts obtained from WT, AMPEL1^Tg/+^, Peli1^−/−^, and CP1KO mice 24 h post-CLP surgery. (**A**) Representative 4-HNE-stained images of the myocardium obtained from WT, AMPEL1^Tg/+^, Peli1^−/−^, and CP1KO mice 24 h post-CLP surgery; (**B**) representative Bax-stained images of hearts obtained from WT, AMPEL1^Tg/+^, Peli1^−/−^, and CP1KO mice 24 h post-CLP surgery; (**C**) representative image showing TUNEL-positive cells in hearts obtained from WT, AMPEL1^Tg/+^, Peli1^−/−^, and CP1KO mice 24 h post-CLP surgery; (**D**) graphical representation of myocardial apoptotic cell death in WT, AMPEL1^Tg/+^, Peli1^−/−^, and CP1KO mice 24 h post-CLP surgery. Values are mean ± S.E.M. (*n* = 6). Where ns-not significant; * *p* < 0.05.

Figure 9A shows expression of 4-HNE in WT, AMPEL1^Tg/+^, CP1KO, and Peli1^−/−^ mice 24-h after CLP surgery. Decreased levels of 4-HNE expression were observed in overexpressed transgenic Peli1 mice (AMPEL1^Tg/+^CLP) compared to WTCLP; however, Peli1^−/−^CLP and CP1KOCLP showed a significant increase in 4-HNE expression when compared to their WTCLP counterparts.

### 3.4. Assessment of Cardiac Myocyte Apoptosis at 24 Hours after Severe Sepsis 

Figure 9C,D show the results of a TUNEL assay performed in cardiac tissues at the 24-h time point after sepsis surgery. Overexpression of Peli1 (AMPEL1^Tg/+^) in mice led to a significant reduction of TUNEL-positive cells compared to the Peli1^−/−^ (4.69 ± 2.29 vs. 21.70 ± 6.71; *n* = 6; *p* = 0.0002), CP1KO (4.69 ± 2.29 vs. 15.27 ± 3.02; *n* = 6; *p* = 0.0097), and WTCLP (4.69 ± 2.29 vs. 11.67 ± 6.90; *n* = 6; *p* = 0.04) mice subjected to sepsis. Figure 9C clearly demonstrates elevated numbers of apoptotic cells in Peli1^−/−^ group of mice compared to the WTCLP group (21.70 ± 6.71 vs. 11.67 ± 6.90; *n* = 6; *p* = 0.03). However, there was no difference between the WTCLP and CP1KOCLP groups (11.67 ± 2.82 vs. 15.27 ± 3.02; *n* = 6; *p* = 0.41). 

The extent of apoptosis (demonstrated via TUNEL-positive cells) was further correlated with immunofluorescence expression of Bax protein (Figure 9B) in these experimental groups at 24 h post-CLP. A significant decrease in the expression level of Bax protein was seen in the cardiac AMPEL1^Tg/+^CLP mice compared to the WTCLP, Peli1^−/−,^ and CP1KO sepsis groups. Figure 9B also clearly demonstrates increased expression of Bax protein in the Peli1^−/−^ and CP1KO groups compared to the WTCLP group. Bax protein expression significantly correlates with the extent of TUNEL-positive cells in the CLP groups. (Images were taken, and observation results were carried out by double-blind study).

### 3.5. Assessment of Pro-Inflammatory Cytokines following Severe Sepsis 

**Figure 10:**
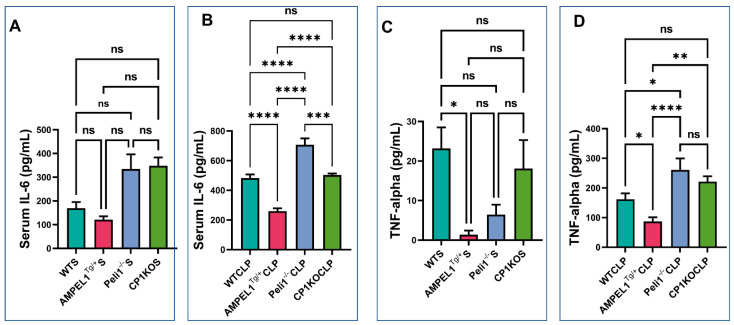
Graphical representation of serum inflammatory cytokine concentration comparing wild-type sham (WTS), AMPEL1^Tg/+^(S), and CLP; Peli1^−/−^ sham and CLP; and CP1KOS and CLP: (**A**) serum IL-6 concentration (pg/mL) at 6 h post-sham surgery, (**B**) serum IL-6 concentration (pg/mL) at 6 h post-CLP surgery, (**C**) serum TNF-α concentration (pg/mL) at 6h post-sham surgery, (**D**) serum TNF-α concentration (pg/mL) at 6 h post-CLP surgery. Values are mean ± S.E.M. (*n* = 6). Where ns-not significant; * *p* < 0.05, ** *p* < 0.01, *** *p* < 0.001, **** *p* < 0.0001.

In the suggested study groups, we measured the serum levels of pro-inflammatory cytokines TNF-α and IL-6 at 6 h post-CLP procedure. IL-6 was significantly increased in the serum samples of Peli1^−/−^ (706.8 ± 43 vs. 259.7 ± 18.70; pg/mL; *n* = 6; *p* < 0.0001) and CP1KO (503.3 ± 10.59 vs. 259.7 ± 18.70; pg/mL; *n* = 6; *p* < 0.0001) sepsis groups when compared to the AMPELl^Tg/+^ group. Interestingly, we found that the Peli1 global knockout group showed more IL-6 released in the blood stream when compared to the WTCLP (706.8 ± 43 vs. 483.2 ± 24.01; pg/mL; *n* = 6; *p* < 0.0001) and CP1KO sepsis groups (706.8 ± 43 vs. 503.3 ± 10.59; pg/mL; *n* = 6; *p* = 0.0002). However, there was no difference in serum IL-6 level between the WTCLP and CP1KO sepsis groups (483.2 ± 24.01 vs. 503.3 ± 10.59; pg/mL; *n* = 6; *p* = 0.95) (Figure 10B).

Similarly, serum TNF-α level was found to be significantly reduced in the AMPEL1^Tg/+^ sepsis group compared to the WTCLP (86.89 ± 14.36 vs. 161.8 ± 20; pg/mL; *n* = 6; *p* = 0.04), Peli1^−/−^ (86.89 ± 14.36 vs. 260.4 ± 39.04; pg/mL; *n* = 6; *p* < 0.0001), and CP1KO (86.89 ± 14.36 vs. 221.4 ± 17.77; pg/mL; *n* = 6; *p* = 0.0010) sepsis groups. Elevated levels of TNF-α were observed in the Peli1^−/−^ global knockout sepsis group compared to the WTCLP group (260.4 ± 39.04 vs. 161.8 ± 20; pg/mL; *n* = 6; *p* = 0.01), but no difference was found between the WTCLP and CP1KO groups (161.8 ± 20 vs. 221.4 ± 17.77; pg/mL; *n* = 6; *p* = 0.10) (Figure 10D). From our data, it is very obvious that both IL-6 and TNF-α levels were significantly elevated in Peli1 global knockout mice after sepsis compared to the other sepsis groups. Therefore, the severity is greater when Peli1 is globally knocked out and subjects are exposed to sepsis compared to CP1KO, AMPEl1^Tg/+^, and even WTCLP.

In our present study, there is no significant change in these circulating inflammatory markers in sham surgery involving AMPEL1^Tg/+^, Peli1 KO, and CP1KO mice compared to their wild-type sham (WTCLP) controls (Figure 10A,C). However, only TNF-α level was found to be significantly lower in the AMPEL1^Tg/+^ sham (baseline) group compared to WTS (1.42 ± 1.03 vs. 23.18 ± 5.34; pg/mL; *n* = 4; *p* = 0.03) (Figure 10C).

## 4. Discussion

Our previous studies indicated that Peli1 is an important angiogenic molecule that plays a significant role downstream from the VEGF/Flk-1 system to induce neovascularization and increase blood flow in MI and HLI models [11,12,13]. Recent studies documented important primary actions of Peli1 on the regulation of Toll-like receptors (TLRs) signaling and immunoregulatory functions and inflammation [9,20,21,22]. Molecular signaling pathways are generally controlled by phosphorylation of proteins, but these pathways are also regulated by ubiquitination [23]. Our new finding shows Peli1 acts as a negative regulator of inflammatory responses as observed by reduced TNF-α and IL-6 levels in Peli1-overexpressed mice (AMPEL1^Tg/+^) when compared to the corresponding WT controls and Peli1^−/−^ and CP1KO mice subjected to CLP surgery. This present observation tallies with the study by Marsh et al. [24], who reported that Peli1 is expressed in the airways of normal subjects and in those with chronic obstructive pulmonary disease (COPD). They also suggested that Peli1 regulates TLR3 signaling in response to viral infection. In particular, they [24] observed increased levels of proinflammatory cytokines IL-6 and TNFα in Peli1 knockout mice upon viral infection. The results of our present study have also provided new information about how innate immunity relates to the TLR pathway and is regulated by Peli1. Each Pellino family member carries E3 ligase activity, is a substrate for IRAK kinases, and is able to interact with signaling molecules [25,26,27,28,29]. We need to explore and understand the molecular mechanisms that control Peli1 functions in various experimental situations. We believe that the role of Peli1 and the inflammatory response observed depends entirely on the type of infection. The pathophysiology of sepsis has a significant effect on cardiac performance, leading to direct cardiac dysfunction and eventually mortality if untreated. We demonstrated that cardiac-specific or global knockout of Peli1 individually induced pro-inflammatory and pro-apoptotic responses in sepsis, which contributed to myocardial inflammation, cell death, and overall cardiac dysfunction. However, there was a remarkable reduction of pro-inflammatory cytokines and pro-apoptotic protein (Bax), followed by rescue of cardiac function in the same CLP model using Peli1-overexpressed mice. 

The present study provides engrossing evidence of the cardioprotective role of Peli1-E3 ubiquitin ligase during severe intra-abdominal sepsis. Pellino-1 deficiency has proven to induce multiple detrimental physiological effects that include: (1) atherogenic immune cell activation (Th1 and CD4 cells), (2) elevated systemic pro-inflammatory cytokines (IL-6 and TNF-α), (3) increased levels of circulating IgE and IgG2a [30], and (4) decreased angiogenic response during myocardial infarction [11,12]. Previous studies have revealed cardiac dysfunction during sepsis increases mortality in affected patients [14,16,31,32]. The multifactorial nature of this cardiac dysfunction continues to limit the ability of clinicians to effectively identify and treat patients with sepsis and/or multi-organ dysfunction. Given these therapeutic hardships, which persist despite ongoing advances in critical care, our exploration of Peli1 serves to identify a potential therapeutic approach to blunt the widespread, deleterious effects of this disease process. 

Inflammatory cytokines and oxidative stress are among the many cardiac depressants that are known to contribute to the cardiac dysfunction seen with sepsis [33,34,35]. Within our experiment, ELISA analysis displayed how Peli1 overexpression led to a decrease in circulating inflammatory cytokines. Conversely, when Peli1 was knocked out (both globally and in cardiomyocytes alone), there was an increase in inflammatory cytokines, suggesting a potential role for Peli1 in mitigating the inflammatory cascade present in sepsis. In our previous study, we found overexpression of Peli1 led to the inhibition of inflammatory lymphocytes and an overall reduction in the inflammatory response that accompanies ischemia [13]. The cellular hypoxia induced by sepsis is believed to drive the multi-organ dysfunction that can occur if this condition follows its natural course. The increased neovascularization that accompanies Peli-1 overexpression offers a potential therapeutic strategy to combat this global tissue hypoxia. 

Cardiac apoptosis 24 h after CLP surgery demonstrated a protective role of Peli1 protein in the AMPEL1^Tg/+^ group compared to all other experimental groups. The proportion of TUNEL-positive cells varied according to Peli1 expression, with overexpression (AMPEL1^Tg/+^) leading to a significant reduction and Peli1 gene knockout (Peli1KO and CP1KO) leading to a significant increase in TUNEL-positive cells. The improved cellular survival associated with Peli1 overexpression was again shown with the reduction of 4-Hydroxy-2-Nonenal (4-HNE) staining. 4-HNE, a byproduct of lipid peroxidation, was significantly decreased when Peli1 was overexpressed and significantly increased in both knockout models. 4-HNE is a highly toxic aldehyde product of lipid peroxidation and is a highly sensitive marker of oxidative damage.

Echocardiography remains the cornerstone for the diagnosis of septic cardiomyopathy, as it continues to be highly utilized in the hemodynamically unstable, critically ill patient. Initially, left ventricular ejection fraction (LVEF) was the sole parameter used to make this diagnosis, which led to this condition being underreported given its poor sensitivity and specificity [3,31,36,37,38]. Reliance on this parameter alone also offers insight into the paradoxical increase in survival that was seen in septic patients with decreased LVEF in a study by Parker et al. [39]. Patients with profound shock have diminished peripheral vascular resistance due to vasoplegia, which can lead to a pseudo-normalization of their EF. Conversely, adequate fluid resuscitation and the use of vasopressors in these patients may account for the discordance between decreased EF and mortality [40]. This phenomenon was seen in our study at the 24-h time point in CP1KO mice that underwent CLP. We also noted a significantly decreased left ventricular internal diameter in systole within this group at the same time point. This decrease in intracardiac volume during a systemic septic response can further explain the observed pseudo-normalization of EF seen in this particular group. Overall echo results from three different genetically modified mice (AMPEL1^Tg/+^, Peli1^−/−^, and CP1KO) 24 h post-CLP surgery showed several exciting findings. AMPEL1^Tg/+^ mice showed increased EF and FS compared to WTCLP mice, whereas Peli1^−/−^ mice showed the opposite results, i.e., significantly decreased EF and FS in the Peli1^−/−^CLP group compared to their WTCLP counterparts. Surprisingly, EF and FS were significantly increased in CP1KO mice after surgery compared to the WTCLP group. Left ventricular internal diameter at both systole and diastole showed no difference in both AMPEL1^Tg/+^ and Peli1^−/−^ mice compared to the respective WT groups at 24 h post-CLP. However, in the CP1KO mice, there was a notably significant difference between the WTCLP and CP1KO groups at 24 h post-surgery. 

There was no difference in LVVOL at both systole and diastole in the AMPEL1^Tg/+^ and Peli1^−/−^ mice in the CLP groups at 24 h post-surgery. However, CP1KO mice showed significantly decreased LV volumes at both systole and diastole in the CP1KOCLP group compared to the WTCLP at 24 h after surgery. The measured heart rate showed no difference in all the genetically modified mice between the WT and CLP groups post-operatively. Wall thickness measured with both LV anterior and posterior walls showed no notable differences between the CLP groups in both AMPEL1^Tg/+^ mice and Peli1^−/−^ mice. However, CP1KO mice showed significantly increased wall thickness in both anterior and posterior walls, mimicking hypertrophic conditions. This increase could be due to significant edema in the ventricular walls or other unknown reasons. Stroke volume was not affected in AMPEL1^Tg/+^ and Peli1^−/−^ mice after CLP surgery compared to their respective WT group.

Finally, cardiac output was significantly increased in AMPEL1^Tg/+^ mice and significantly decreased in CP1KO mice at 24 h post-CLP, whereas Peli1^−/−^ mice showed a reduction but not a statistically significant difference between the CLP groups at the same time interval. CP1KO mice at 24 h post-CLP had increased EF and FS, reduced LV volumes and LV internal diameters, increased wall thickness in both anterior and posterior wall, and decreased SV and CO. All these results with CP1KO mice show that there is a significant cardiac dysfunction even though EF and FS were high, mimicking acute heart failure with preserved EF (Figure 8). Correlating with these results, we also observed significant mortality in CP1KO mice post-CLP compared to WT mice. It is unclear why CP1KO mice appear to have more significantly hampered cardiac function in sepsis relative to Peli1 total body knockout mice.

The aforementioned findings can in part be explained by the Peli1 protein’s ability to mitigate oxidative stress on a cellular level, a finding our lab has previously shown [11,12,18] in the context of myocardial infarction and hind limb ischemia. The systemic inflammatory response (cytokine storm) seen during sepsis drives the underlying tissue ischemia and resultant multi-organ dysfunction. Based on our findings in this experiment, the pro-angiogenic potential of Peli 1 could account for a mitigated cellular hypoxia and therefore a less severe response to an infectious insult. This experiment shows the importance of the Peli1 protein in this cascade as AMPEL mice mounted a superior response to a septic insult as displayed by preserved cardiac function, decreased apoptosis, markers of oxidative stress, and deleterious inflammatory cytokines. 

In conclusion, Peli1 has the potential to attenuate the sepsis induced myocardial dysfunction and oxidative stress that accompanies severe sepsis. Transgenic overexpression of this gene leads to a reduction in 4-HNE (a marker of oxidative stress), TUNEL-positive cells (a marker for cells undergoing apoptosis), and the inflammatory cytokines IL-6 and TNF-α. This suggests that Peli 1 expression mitigates the oxidative stress and inflammatory response seen during severe sepsis. In this mouse population, echocardiography also demonstrated preserved cardiac function suggesting its potential cardio-protective role. Pellino-1 may function as a novel therapeutic target to ameliorate the deleterious inflammatory cascade, oxidative stress, and cardiac suppression that accompanies severe sepsis. However, a further detailed study is required with an extended timeline related to sepsis with Peli1 treatment to better understand its in-depth molecular mechanism.

## Data Availability

Data will be made available on reasonable request.
